# Supplementary material for: Development of a novel, pan-variant aerosol intervention for COVID-19
Source: bioRxiv. 2021 Sep 14:2021.09.14.459961. Preprint. [Version 1] doi: 10.1101/2021.09.14.459961 (PMC8452093; doi:10.1101/2021.09.14.459961)

bioRxiv preprint doi: https://doi.org/10.1101/2021.09.14.459961; this version posted September 14, 2021. The copyright holder for this preprint (which was not certified by peer review) is the author/funder. This article is a US Government work. It is not subject to copyright under 17 USC 105 and is also made available for use under a CC0 license.

Supplemental Materials

Supplemental Table 1. ACE2 binding to SARS-CoV-2 Spike protein is not significantly altered after nebulization. All samples from trials 1-4 were normalized to 27 µg/ml according to R&D Systems ELISA measurements and assayed for binding to the SARS-CoV-2 Spike RBD. Data are recorded as EC50 values in ng/ml with 95% confidence intervals. The differences between Pre-Nebulization and Post-Nebulization binding activity are indicated as a % change for each APN01 dilution. In this Table, Pre-Nebulization corresponds to the Control samples in Figures 3 and 4 of the main text.

	Sample	EC50	95% CI
Trial 1 (2.5 mg/ml)	Pre-Nebulization	58.41	40.56-85.50
	Unnebulized Volume	41.08	34.85-48.60
	Post-Nebulization	42.62	33.18-55.24
	% change Pre to Post	-27.03	
Trial 2 (2.5 mg/ml)	Pre-Nebulization	44.62	39.42-50.58
	Unnebulized Volume	43.65	37.55-50.85
	Post-Nebulization	39.81	34.60-45.86
	% change Pre to Post	-10.78	
Trial 3 (0.1 mg/ml)	Pre-Nebulization	57.32	43.41-76.55
	Unnebulized Volume	50.05	44.29-56.70
	Post-Nebulization	55.82	48.18-64.85
	% change Pre to Post	-2.62	
Trial 4 (0.1 mg/ml)	Pre-Nebulization	43.81	38.53-49.89
	Unnebulized Volume	34.94	32.28-37.79
	Post-Nebulization	43.75	35.38-54.33
	% change Pre to Post	-0.14	

bioRxiv preprint doi: https://doi.org/10.1101/2021.09.14.459961; this version posted September 14, 2021. The copyright holder for this preprint (which was not certified by peer review) is the author/funder. This article is a US Government work. It is not subject to copyright under 17 USC 105 and is also made available for use under a CC0 license.

Supplemental Table 2. APN01 enzymatic function remains unaltered after nebulization. Data from trials 1-4 were recorded as (ΔRFU/min)/ng for the three starting dilutions of APN01 at 25 ng/ml, 50 ng/ml, 100 ng/ml. The difference between Pre-Nebulization and Post-Nebulization enzymatic activity is indicated as a % change for each APN01 dilution and the mean change and standard deviation across all three dilutions. In this Table, Pre-Nebulization corresponds to the Control samples in Figures 3 and 4 of the main text.

		APN01 Starting Dilutions in assay				
		25 ng/mL	50 ng/mL	100 ng/mL		
	Sample	Units (ΔRFU/min)/ng			mean	StDev
Trial 1 (2.5 mg/ml)	Pre-Nebulization	81.74	75.93	68.58		
	Unnebulized Volume	77.54	74.26	69.09		
	Post-Nebulization	80.18	72.85	69.95		
	% change Pre to Post	-1.91	-4.06	2.00	-1.32	3.07
Trial 2 (2.5 mg/ml)	Pre-Nebulization	97.39	81.94	72.96		
	Unnebulized Volume	94.69	84.27	76.88		
	Post-Nebulization	101.59	87.99	79.41		
	% change Pre to Post	4.31	7.38	8.84	6.85	2.31
Trial 3 (0.1 mg/ml)	Pre-Nebulization	93.14	76.08	70.67		
	Unnebulized Volume	95.78	82.34	74.60		
	Post-Nebulization	97.59	79.81	73.27		
	% change Pre to Post	4.78	4.90	3.68	4.45	0.67
Trial 4 (0.1 mg/ml)	Pre-Nebulization	99.76	83.16	76.42		
	Unnebulized Volume	103.01	91.98	83.35		
	Post-Nebulization	104.70	87.48	73.27		
	% change Pre to Post	4.95	5.19	-4.12	2.01	5.31

bioRxiv preprint doi: https://doi.org/10.1101/2021.09.14.459961; this version posted September 14, 2021. The copyright holder for this preprint (which was not certified by peer review) is the author/funder. This article is a US Government work. It is not subject to copyright under 17 USC 105 and is also made available for use under a CC0 license.

Supplemental Table 3. Toxicology parameters studied*.

A. Test Atmospheres

B. Inhaled Dose

C. Mortality and Clinical Signs

D. Body Weights and Body Weight Changes

E. Food Consumption

F. Clinical Pathology

G. Heart Rate and Blood Pressure

H. Ophthalmic Examination

I. Electrocardiography

J. Respiratory Function

K. Peripheral and Venous Blood Oxygen Saturation and Blood pH

L. Functional Observational Battery

M. Serum Drug Levels and Toxicokinetics

N. Organ Weights

O. Gross Pathology and Histopathology

*This study was conducted under Good Laboratory Practices. The table lists the parameters studied. Data for A, B and M are cited in the text. Detailed data (available on request) for the other parameters did not reveal treatment-related abnormalities.

bioRxiv preprint doi: https://doi.org/10.1101/2021.09.14.459961; this version posted September 14, 2021. The copyright holder for this preprint (which was not certified by peer review) is the author/funder. This article is a US Government work. It is not subject to copyright under 17 USC 105 and is also made available for use under a CC0 license.

Supplemental Figure 1. HPLC chromatogram illustrating quantitation of APN01 extracted from filters placed in the nebulized atmosphere at 0.075 mg/L APN01. Absorbance Units (AU) monitored at 220 nm are plotted as a function of time. Chromatography conditions are described in Materials and Methods. APN01 was resolved as a single peak eluting between five and six minutes. The concentrations of test article in the processed filter samples were determined from each sample's peak area using the linear regression parameters derived from the calibration curves and correcting the resulting concentration by multiplying by the appropriate dilution factor, as applicable. In addition to supporting quantitation of APN01 in the nebulized atmosphere, this result supports maintenance of the physical integrity of APN01 during the process of aerosolization.

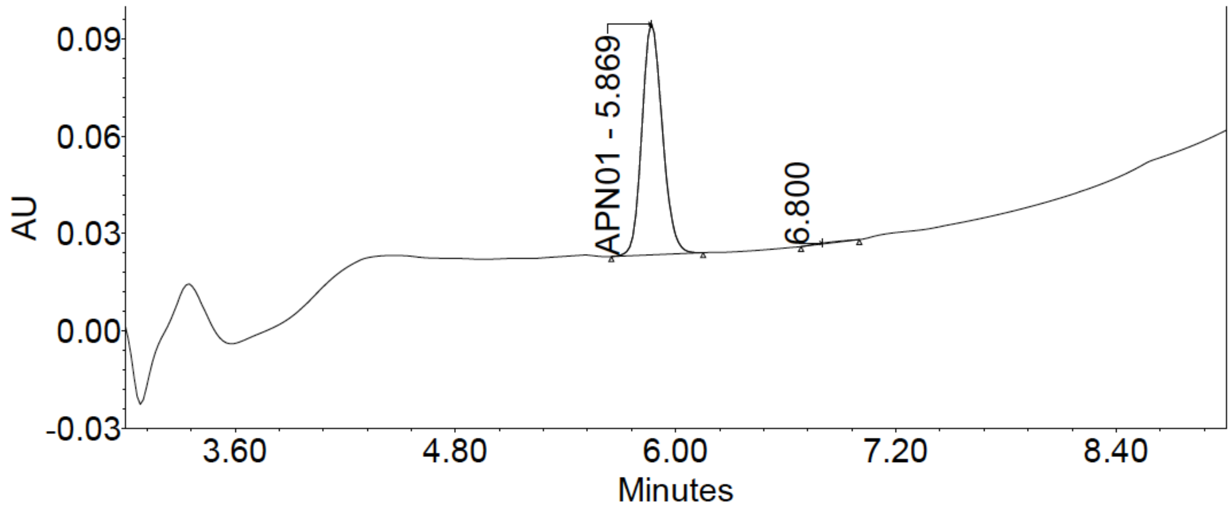

Supplement: Supplementary file 1 [file NIHPP2021.09.14.459961V1-supplement-1.pdf]
